# Gun Violence Trends in US Cities During the Early Phase of the COVID-19 Pandemic

**DOI:** 10.1001/jamanetworkopen.2024.54760

**Published:** 2025-01-16

**Authors:** Chandler Hall, Nick Wilson, Alex R. Piquero

**Affiliations:** 1 Center for American Progress; 2 University of Miami, Coral Gables, Florida

## Abstract

This cross-sectional study evaluates whether there was any variation in gun violence trends in US cities during the early phase of the COVID-19 pandemic.

## Introduction

It has been well established that the United States experienced an unprecedented increase in gun violence during the COVID-19 pandemic.^1^ Studies also showed significant demographic differences in the risk of firearm homicide.^2^ The objective of this study was to examine whether there was variation in gun violence trends in US cities during the early phase of the COVID-19 pandemic, from March 15, 2020, to June 30, 2021.

## Methods

This cross-sectional study used incident data of 32 238 fatal and nonfatal firearm injuries from the Gun Violence Archive from the first week after the World Health Organization^3^ declared COVID-19 a pandemic, March 15, 2020, to June 30, 2021, at which point crime data show homicides began to steadily decrease, to explore developmental trajectories of gun violence among 88 US cities of at least 250 000 residents.^4^

This study uses the group-based trajectory method, a type of finite mixture modeling designed to identify distinct subgroups that follow similar patterns of behavior over time.^5^ To measure the marginal change in a city’s trajectory during the sample period and account for seasonality, incident data for each city included in the sample are aggregated to 4-week rolling averages of fatal and nonfatal firearm injuries and take the difference compared with 2019’s 4-week rolling average. Model selection was determined using the bayesian information criterion (BIC). We followed the Strengthening the Reporting of Observational Studies in Epidemiology (STROBE) reporting guideline. Analyses were completed between April and October 2024 in R statistical software version 4.3.1 (R Project for Statistical Computing). The eMethods in Supplement 1 provides additional methodological details.

## Results

**Figure 1.**
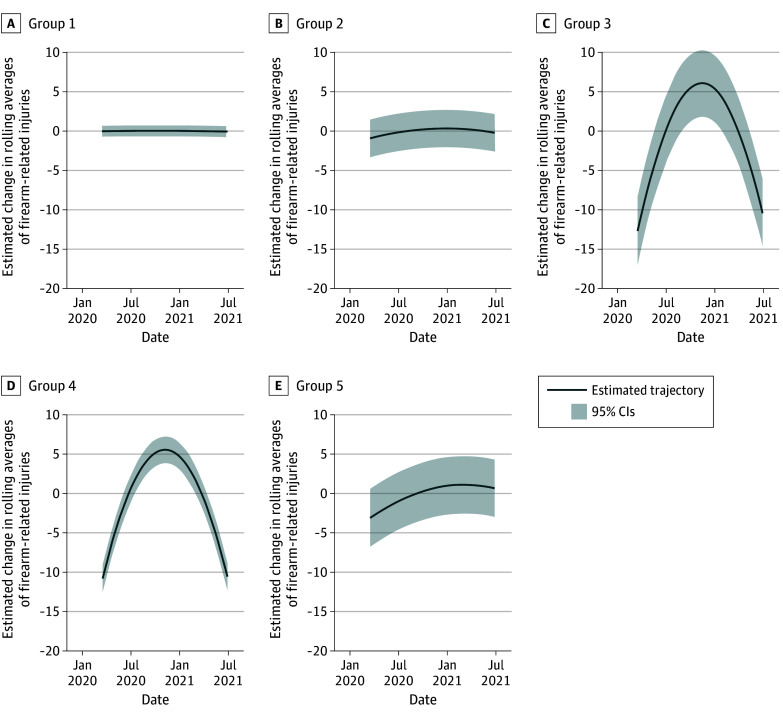
Group-Based Trajectory Model Estimates

**Figure 2.**
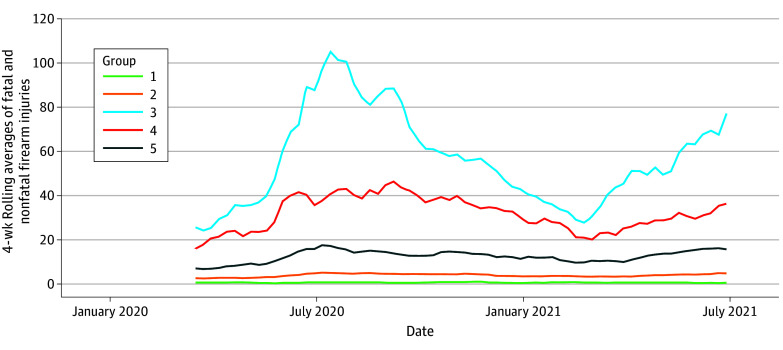
Fatal and Nonfatal Firearm Injuries by Group Assignment

Figure 1 shows that the model identified 5 distinct trajectories (BIC = 24 162.19): (1) a group of 23 cities with an average posterior probability of assignment (APPA) of 0.999; (2) a group of 42 cities with an APPA of 0.996, (3) a group of 2 cities with an APPA of 1.00, (4) a group of 2 cities with an APPA of 1.00, and (5) a group of 19 cities with an APPA of 0.999. Notably, group 3, which only included Chicago, Illinois, and New York, New York, saw the largest increase in their developmental trajectory from March 15, 2020, to June 30, 2021, experiencing an estimated 3491 additional firearm-related injuries combined compared with March 15, 2018, to June 30, 2019. Figure 2 shows how the 5 trajectories varied over time with respect to fatal and nonfatal firearm injuries.

## Discussion

This study finds variation in both the timing and surge of gun violence between cities during the COVID-19 pandemic. Use of data from the Gun Violence Archive is a necessity at this stage given the lack of a comprehensive federal fatal and nonfatal firearm data collection system but is limited because it collects data from public records and public media and may suffer from some systematic biases.^6^ Study findings have implications for understanding the factors associated with the variability across cities over time, which should spur additional causal research to inform context-specific policy responses such as examining the demographic variability of the changes observed across and between cities.

## Notes

Supplement 1. **eMethods**.
eReferences.

Supplement 2.
Data Sharing Statement
