# Supplementary material for: Gun Violence Trends in US Cities During the Early Phase of the COVID-19 Pandemic
Source: JAMA Netw Open. 2025 Jan 16;8(1):e2454760. doi: 10.1001/jamanetworkopen.2024.54760 (PMC11739989; doi:10.1001/jamanetworkopen.2024.54760)
Supplement: Supplement 1. — eMethods. eReferences. [file jamanetwopen-e2454760-s001.pdf]

Supplemental Online Content

Hall C, Wilson N, Piquero AR. Gun violence trends in US cities during the early phase of the COVID-19 pandemic. *JAMA Netw Open*. 2025;8(1):e2454760. doi:10.1001/jamanetworkopen.2024.54760

eMethods.

eReferences.

This supplemental material has been provided by the authors to give readers additional information about their work.

© 2025 Hall C et al. *JAMA Network Open*.

eMethods

For over thirty years, researchers have used group-based trajectory modeling (GBTM) for analyzing developmental trajectories, e.g., the evolution of an outcome over time, across many different disciplines studying many different outcomes including, for example, criminal behavior within birth cohorts, distribution of crime at street segments over time, internet usage, rankings of Formula 1 drivers, and so forth (Nagin, 2014). This methodology has become a valuable tool for identifying and summarizing complex patterns of behavior over time.

Data required for the use of GBTM are longitudinal data with a time dimension. Although there are other approaches to modeling behavior over time, these methods focus on individual variability about a population trend, whereas GBTM assesses the extent to which there are meaningful subgroups within the overall population that follow distinct developmental trajectories, such as the case in the current study where we are interested in modeling unique patterns of gun violence across many different cities that can be summarized into unique groups that follow similar patterns. This methodology was first introduced by Nagin and Land (1993) for the analysis of crimes over the life-course in a large sample of individuals.

Specific information regarding the model estimation procedures is beyond the scope of this supplement, but is readily available elsewhere (Nagin, 2005). As noted by Nagin (2014, 208), GBTM "assumes individual differences in trajectories can be summarized by a finite set of different polynomial functions of age or time." Each set of trajectory groups contains units of observation that are assigned to a specific group based on their probability of belonging to that group relative to other groups. And units of observation in one group tend to resemble one another more than they do other units of observation in another group. Model estimates also produce output that contains the average probability of group membership, with values >.7 being acceptable. A final and quite useful piece of output from GBTM is the visual display which plots how the groups trend over time with respect to the outcome of interest.

Taking our specific question, say two cities had very high and steady rates of gun violence over time, three cities had very high but briefly contained rates of gun violence, and five cities had almost no gun violence, traditional models would compromise at an average estimate, but the GBTM would visually show that there are groups of different cities that have different values and shapes of gun violence over time.

The group-based trajectory modeling uses the quadratic form $Y_{it} = a + B_1 Rolling\ Weekly\ Average_t + Rolling\ Weekly\ Average\ _t^2$

© 2025 Hall C et al. *JAMA Network Open*.

to estimate each group's trend over the sample period, where Y_{it} is the predicted 4-week rolling average of gun violence incidents for city *i* in period *t*. To account for possible bias because of city size and different levels of gun violence across cities, the rolling weekly averages are centered prior to modeling, expressed as $Y_{it} = Y_{it} - \bar{Y}_i$. The final GBTM model estimates found in Supplemental Table 1 contain city assignments across the five trajectory groups.

To test our research question, the sample period was chosen based on COVID-19 being declared a pandemic by the World Health Organization on March 11th. Similarly, based on FBI crime data, the national homicide rate continued to spike during the early phase of the pandemic through June of 2021, after which, homicide rates have steadily declined after accounting for seasonal cycles.

eReferences

Nagin DS. *Group-based modeling of development.* Cambridge: Harvard University. Press; 2005.

Nagin DS, Land KC. Age, criminal careers, and population heterogeneity—specification and estimation of a nonparametric, mixed poisson model. *Criminology.* 1993;31:327–362. doi: 10.1111/j.1745-9125.1993.tb01133.x.

Nagin DS. Group-based trajectory modeling: an overview. *Ann Nutr Metab*. 2014;65(2-3):205-10. doi: 10.1159/000360229. Epub 2014 Nov 18. PMID: 25413659.

© 2025 Hall C et al. *JAMA Network Open*.

Supplemental Table 1. City Assignments Across Trajectory Groups

Group 1	Group 2	Group 3	Group 4	Group 5
Anaheim, California	Albuquerque, New Mexico	Chicago, Illinois	Philadelphia, Pennsylvania	Atlanta, Georgia
Anchorage, Alaska	Aurora, Colorado	New York, New York	Saint Louis, Missouri	Baltimore, Maryland
Arlington, Texas	Austin, Texas			Buffalo, New York
Chandler, Arizona	Bakersfield, California			Cleveland, Ohio
Chula Vista, California	Boston, Massachusetts			Columbus, Ohio
Corpus Christi, Texas	Charlotte, North Carolina			Detroit, Michigan
Fort Wayne, Indiana	Cincinnati, Ohio			El Paso, Texas
Gilbert, Arizona	Colorado Springs, Colorado			Houston, Texas
Henderson, Nevada	Dallas, Texas			Indianapolis, Indiana
Honolulu, Hawaii	Denver, Colorado			Jacksonville, Florida
Irvine, California	Durham, North Carolina			Los Angeles, California
Irving, Texas	Fort Worth, Texas			Memphis, Tennessee
Laredo, Texas	Fresno, California			Miami, Florida
Lincoln, Nebraska	Greensboro, North Carolina			Milwaukee, Wisconsin
Lubbock, Texas	Jersey City, New Jersey			Minneapolis, Minnesota
Madison, Wisconsin	Kansas City, Missouri			New Orleans, Louisiana
Mesa, Arizona	Las Vegas, Nevada			Oakland, California
North Las Vegas, Nevada	Lexington, Kentucky			San Antonio, Texas

© 2025 Hall C et al. *JAMA Network Open*.

Plano, Texas	Long Beach, California			Washington, District of Columbia	
Reno, Nevada	Louisville, Kentucky				
Riverside, California	Nashville, Tennessee				
Saint Petersburg, Florida	Newark, New Jersey				
Santa Ana, California	Oklahoma City, Oklahoma				
	Omaha, Nebraska				
	Orlando, Florida				
	Phoenix, Arizona				
	Pittsburgh, Pennsylvania				
	Portland, Oregon				
	Raleigh, North Carolina				
	Sacramento, California				
	Saint Paul, Minnesota				
	San Diego, California				
	San Francisco, California				
	San Jose, California				
	Seattle, Washington				
	Stockton, California				
	Tampa, Florida				
	Toledo, Ohio				
	Tucson, Arizona				
	Tulsa, Oklahoma				
	Virginia Beach, Virginia				
	Wichita, Kansas				

© 2025 Hall C et al. *JAMA Network Open*.

© 2025 Hall C et al. *JAMA Network Open*.
