# Supplementary material for: Gun Violence Trends in US Cities During the Early Phase of the COVID-19 Pandemic
Source: JAMA Netw Open. 2025 Jan 16;8(1):e2454760. doi: 10.1001/jamanetworkopen.2024.54760 (PMC11739989; doi:10.1001/jamanetworkopen.2024.54760)
Supplement: Supplement 2. — Data Sharing Statement [file jamanetwopen-e2454760-s002.pdf]

Data Sharing Statement

Hall. Gun Violence Trends in US Cities During the Early Phase of the COVID-19 Pandemic. *JAMA Netw Open*. Published January 16, 2025. doi:10.1001/jamanetworkopen.2024.54760

Data
Data available: Yes
Data types: Deidentified participant data, Other (please specify)
Additional Information: Data available from Gun Violence Archive as well as authors upon request.
How to access data: From the authors.
When available: With publication.

Supporting Documents
Document types: None

Additional Information
Who can access the data: Anyone requesting the data.
Types of analyses: Statistical analysis.
Mechanisms of data availability: Upon request.
Any additional restrictions: Attribution to both the Gun Violence Archive (for the original raw data) and to the authors/publication for the group-based trajectory results.
